# Supplementary material for: Single-Cell RNA Sequencing Reveals HIF1A as a Severity-Sensitive Immunological Scar in Circulating Monocytes of Convalescent Comorbidity-Free COVID-19 Patients
Source: Cells. 2024 Feb 6;13(4):300. doi: 10.3390/cells13040300 (PMC10886588; doi:10.3390/cells13040300)
Supplement: Supplementary file 1 [file cells-13-00300-s001.zip › cells-2814219-supplementary.pdf]

a

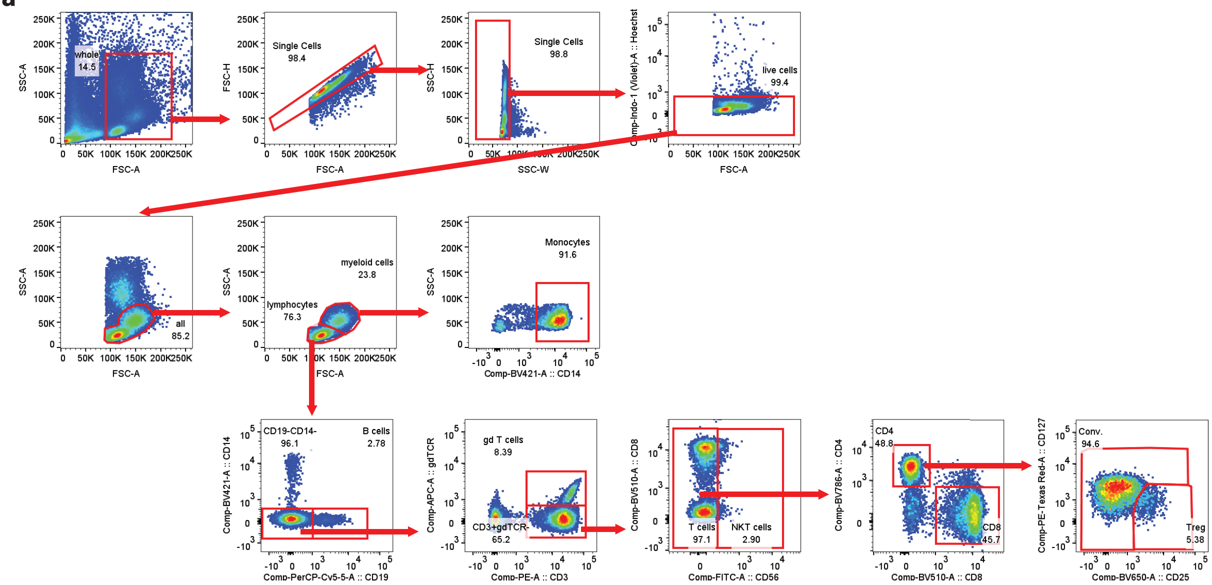

b

Fluorochrome	Antigen	Clone	Comment	Company
BUV737	CD45RA	HI100	564442	BD
BUV395	Hoechst	-	B1155-25MG	SIGMA
BV785	CD4	RPA-T4	300554	Biolegend
BV650	CD25	BC96	302633	Biolegend
BV510	CD8	RPA-T8	301048	Biolegend
BV421 / PB	CD14	HCD14	325616	Biolegend
PerCP-Cy5.5	CD19	HIB19	302230	Biolegend
AF488 / FITC	CD56	MEM-188	304604	Biolegend
PE-Cy7	CCR7 (CD197)	G043H7	353226	Biolegend
TXRed/PE-Dazzle	CD127	A019D5	351336	Biolegend
PE	CD3	SK7	344806	Biolegend
APC	gdTCR	B1	331212	Biolegend

Figure S1. Flow cytometric gating strategy for immune cell subsets.
(a) Cells were selected via FSC-A and FSC-H together with SSC-W and SSC-H; viable cell staining for Hoechst 33258 live/dead dye. Lymphocytes myeloid cells were gated based on FSC-A and SSC-A. Monocytes were identified as CD14^{+} within the myeloid cell population. Within the lymphoid subsets, B cells were identified as CD19^{+} cells; γδ T cells were identified as CD3-γδTCR^{+} cells; NK T cells were identified as CD3^{+}CD56^{+} cells; CD8^{+} gates for CD8 T cell subsets; CD4^{+}CD127^{-}CD25^{+} gates for regulatory T cells; CD4^{+}CD25^{-} gates for CD4^{+} T cell subsets. **(b)** Antibody staining panel used for flow cytometry.

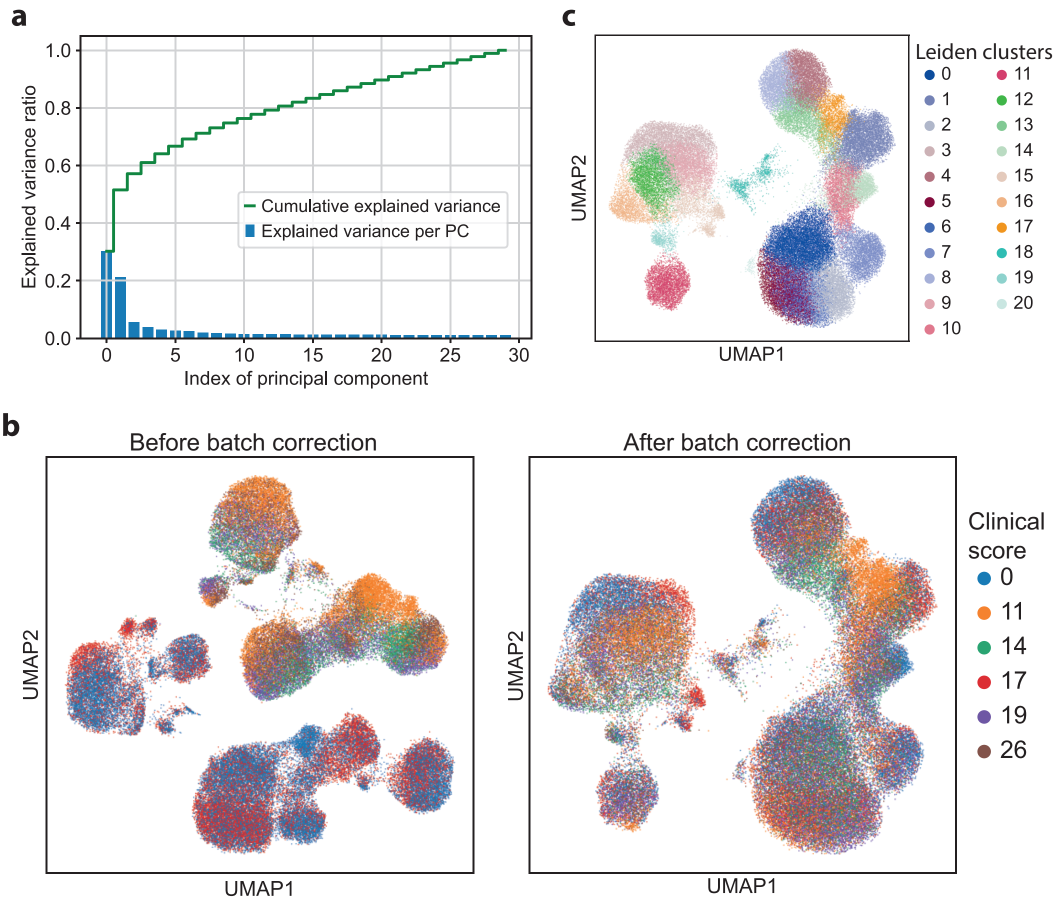

Figure S2. ScRNA-seq data were preprocessed using PCA, UMAP and Leiden clustering.
(a) Explained variance ratio for each principal component (PC) and cumulative explained variance of a principal component analysis with 30 PCs. **(b)** UMAP representation of all cells before (left) and after (right) batch correction for two batches. The first batch consisted of patients with clinical scores of 0 and 17, and the second batch consisted of four patients with clinical scores of 11, 14, 19 and 26. **(c)** UMAP representation of all cells, colored by Leiden clusters calculated with a resolution of 1.5.

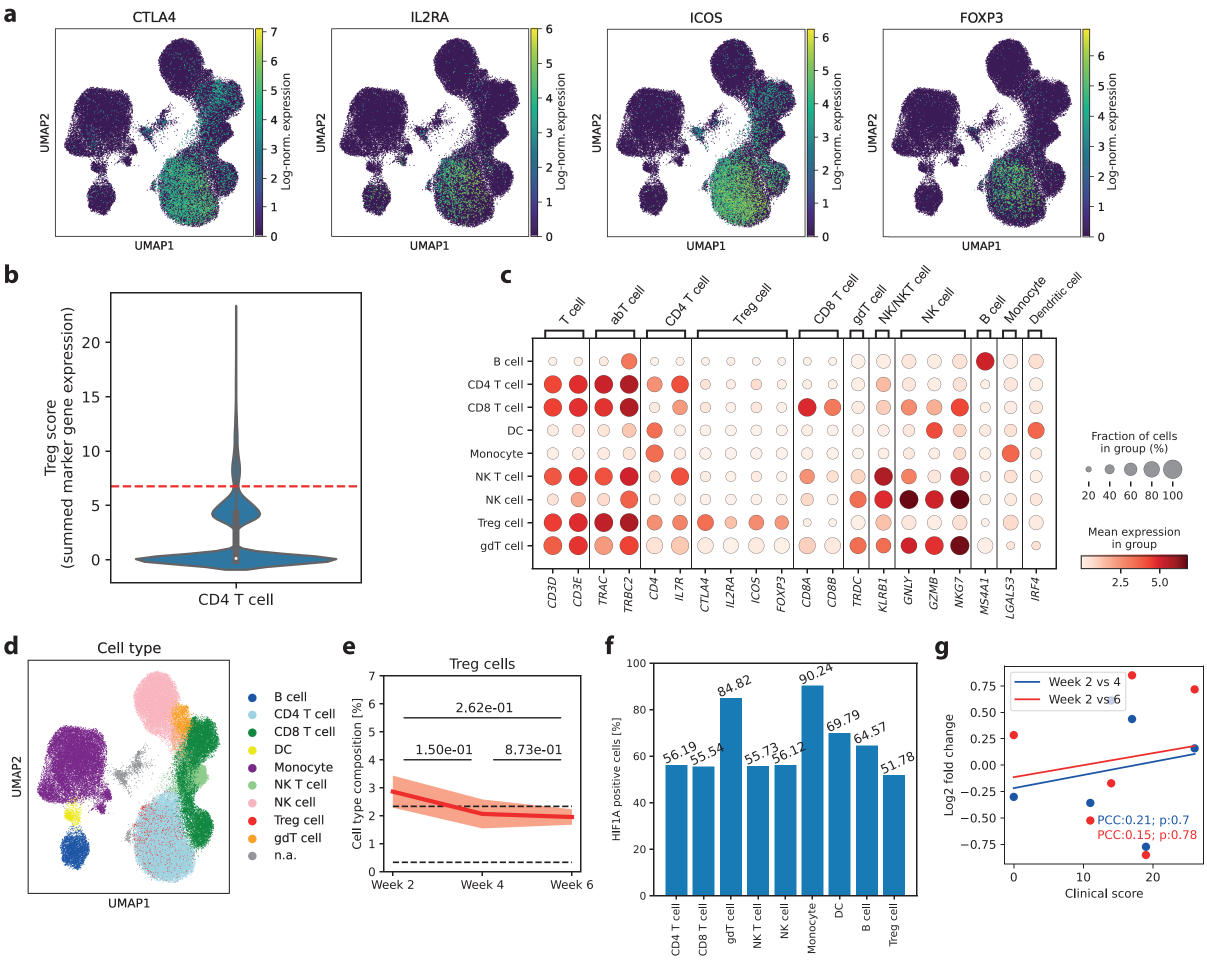

Figure S3. A Treg cell subpopulation is identifiable among the CD4⁺ T cell population but does not exhibit severity-sensitive *HIF1A* expression.

(a) Log-normalized expression of the Treg marker genes *CTLA4*, *IL2RA*, *ICOS*, and *FOXP3* within the UMAP representation of all cells. **(b)** Violin plot depicting the summed-up log-normalized expression of the Treg marker genes *CTLA4*, *IL2RA*, *ICOS*, and *FOXP3* in all CD4⁺ T cells, referred to as Treg score. A red horizontal line is plotted at the threshold we used to annotate the Treg cells, which are expected to exhibit high expression for the four genes. Cells surpassing this Treg score threshold were annotated as Treg cells. **(c)** UMAP representation of all cells, color-coded by cell type, including Treg cells as a subset of the CD4⁺ T cell population. **(d)** Dotplot depicting the marker gene expression for each annotated cell type. **(e)** Relative Treg cell type frequency among all cells over time. The mean relative frequency of all patients ± standard error is shown. For each pair of timepoints, a two-sided Wilcoxon rank sum test was performed. The dashed line indicates the range of physiological variation of the relative cell type frequency for the respective cell type. **(f)** Percentage of cells with *HIF1A* expression greater than zero for each cell type, including Treg cells as a distinct subset from CD4⁺ T cells. **(g)** Log2-fold change (y-axis) of the mean log-normalized *HIF1A* expression in Treg cells in week 2 compared to week 4 (blue) and week 6 (red) depending on the clinical score of the patient (x-axis). The trend of the datapoints is indicated by a linear regression (line). The strength of correlation is described by the Pearson's correlation coefficient (PCC) and p value (p).

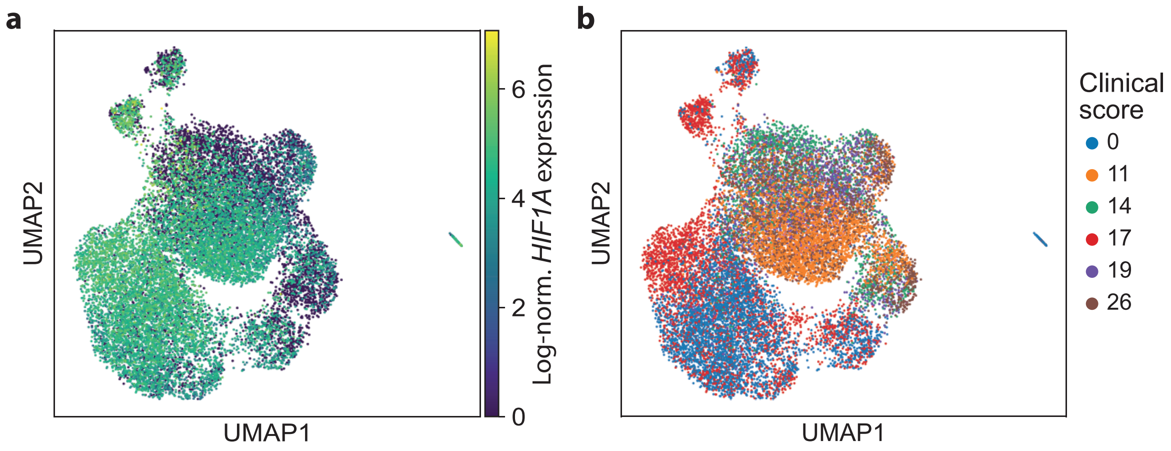

Figure S4. Clustering of the monocyte subpopulation is mainly associated with interindividual differences between patients.

(a) Log-normalized *HIF1A* expression in the UMAP representation of the monocytes after calculating a neighbor graph. **(b)** Patient distribution, represented by the respective clinical score in the UMAP representation of the monocytes after calculating a neighbor graph.

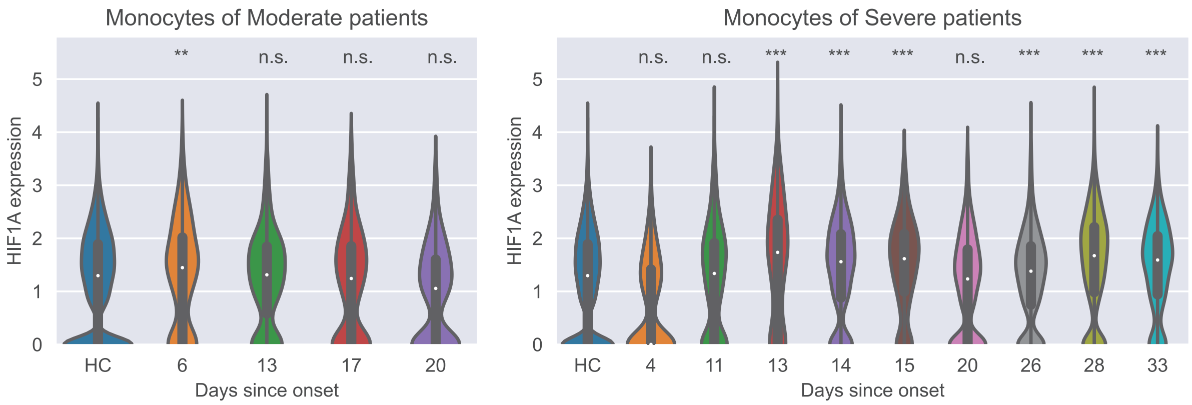

Figure S5. Identification of *HIF1A* as a symptom-severity-sensitive immunological scar is supported by the analysis of a publicly available COVID-19 scRNA-Seq dataset, incorporating a comparison with healthy control patients.

Violin plots indicating *HIF1A* expression in monocytes of COVID-19 patients with a moderate (left) or severe (right) disease course. Each plot includes the expression in healthy controls (HC) on the left. Statistics refer to Wilcoxon Rank Sum Tests with the alternative hypothesis that *HIF1A* expression in healthy controls is lower than in patients at the respective time point (***=p<0.001; **=p<0.01; *=p<0.05; n.s.=p>0.05).

Table S1. Characteristics and symptom scores for the six convalescent COVID-19 patients in our cohort.

Age, hospitalization status, WHO severity score, and our custom clinical score, which is sensitive to severity differences in the mild to moderate range, along with individual symptom scores for each of the six patients in our patient cohort. To calculate our symptom severity score, patients completed a physician-assisted questionnaire in which they assigned scores ranging from 0 to 3 to 15 frequently observed COVID-19 symptoms. A score of 3 indicated the symptom's strongest manifestation. Individual symptom scores were subsequently summed to yield the total clinical score, falling within the range of 0 to 45.

Table S2. The custom primer panel comprises 98 additional genes.

The 98 genes that constitute the custom-designed primer panel, in combination with the predesigned Human T Cell Expression Primer Panel from BD Biosciences, enable an in-depth analysis of immune cell lineage annotations, T cell residency, immune cell metabolism, and immune functions.

Table_S1

Sex	Age (years)	Hospitalisation	WHO score	Total clinical score	Chest pain	Sore throat	Headache	Body aches	Cough	Dyspnea	Fever	Cold	Diarrhea	Ageusia	Anosmia	Weight lost	Sweat	Nausea	Fatigue
**Male**	29	No	1	0	0	0	0	0	0	0	0	0	0	0	0	0	0	0	0
**Male**	65	Yes	4	11	0	0	0	1	1	0	3	0	1	1	0	1	2	0	1
**Female**	65	No	2	14	1	1	0	0	1	0	1	0	0	2	2	1	2	0	3
**Male**	42	Yes	3	17	0	0	2	2	1	2	3	0	0	0	0	2	2	0	3
**Male**	34	No	2	19	1	0	1	0	1	1	1	1	1	3	3	0	3	0	3
**Male**	59	No	2	26	1	1	1	1	3	3	3	0	0	3	3	3	3	0	1

Table_S2

geneID
ABCB1
ABCC1
AHR
ANTXR1
AOC3
AREG
ATXN1
BACH2
BATF
BCL2L1
BIRC3
CAPG
CASP1
CASP12
CASP8
CCL17
CCL22
CD101
CD28
CD34
CD38
CRTAM
CYP1A1
DEPTOR
FABP5
FUT7
GBP1
GCLC
HAVCR1
HIF1A
HSPA1A
ID2

ID3
IFNAR1
IFNAR2
IGF1
IKBKB
IKBKE
IKZF3
IL10
IL1A
IL1B
IRAK4
ITGA1
ITGB1
ITGB7
JAK1
JAML
KLF2
KRT14
MAF
MAP3K7
MAPK1
MET
MS4A1
MTOR
MYD88
NCAM1
NFAT5
NLRP3
NOD1
NOD2
NOTCH1
NR4A1
NRP1
P2RX7

PAG1
PRKCD
PTK7
PYCARD
RARA
RELA
RIPK1
RPS4Y1
RPTOR
RXRA
S100A4
SGK1
SLC2A1
STAM2
STAT2
TAB1
TBK1
TLR4
TLR7
TLR8
TMEM173
TOX
TRAF2
TRAF5
TSLP
TYK2
TYR
VDAC1
XCL2
XIST
ZBTB7B
ZEB2
